# Efficient Multi-View Graph Convolutional Network with Self-Attention for Multi-Class Motor Imagery Decoding

**DOI:** 10.3390/bioengineering11090926

**Published:** 2024-09-15

**Authors:** Xiyue Tan, Dan Wang, Meng Xu, Jiaming Chen, Shuhan Wu

**Affiliations:** College of Computer Science, Beijing University of Technology, Beijing 100124, China; tanxy@emails.bjut.edu.cn (X.T.);

**Keywords:** brain–computer interface, deep learning, motor imagery, graph convolutional networks, self-attention

## Abstract

Research on electroencephalogram-based motor imagery (MI-EEG) can identify the limbs of subjects that generate motor imagination by decoding EEG signals, which is an important issue in the field of brain–computer interface (BCI). Existing deep-learning-based classification methods have not been able to entirely employ the topological information among brain regions, and thus, the classification performance needs further improving. In this paper, we propose a multi-view graph convolutional attention network (MGCANet) with residual learning structure for multi-class MI decoding. Specifically, we design a multi-view graph convolution spatial feature extraction method based on the topological relationship of brain regions to achieve more comprehensive information aggregation. During the modeling, we build an adaptive weight fusion (Awf) module to adaptively merge feature from different brain views to improve classification accuracy. In addition, the self-attention mechanism is introduced for feature selection to expand the receptive field of EEG signals to global dependence and enhance the expression of important features. The proposed model is experimentally evaluated on two public MI datasets and achieved a mean accuracy of 78.26% (BCIC IV 2a dataset) and 73.68% (OpenBMI dataset), which significantly outperforms representative comparative methods in classification accuracy. Comprehensive experiment results verify the effectiveness of our proposed method, which can provide novel perspectives for MI decoding.

## 1. Introduction

A brain–computer interface (BCI) is a system that can directly realize interactive communication between the human brain and the outside world without peripheral nerves and muscles [1]. The research results of BCIs have promoted the development of computer- and mathematics-related disciplines, as well as brain cognitive science and neuroinformatic research, since BCIs have been applied in many fields, such as medical rehabilitation and military practice [2]. The motor imagery (MI) signals generated by the sensorimotor cortex is one of the most focused BCI paradigms by researchers. The event-related desynchronization (ERD)/event-related synchronization (ERS) is mainly induced in the alpha and beta bands accompanied by the phenomenon of spectral oscillation when the users perform MI actions [3]. Researchers identified the parts of the body that generate motor imagery through feature extraction and classification of the collected signals [4].

Methods based on traditional machine learning are commonly used for feature extraction of EEG signals [5]. Machine learning-based methods like Filter Bank Common Spatial Patterns (FBCSP) [6] and Sub-band Common Spatial Pattern (SBCSP) [7] are generally applied the optimal public space filters with category information to extract the spatial distribution of EEG data. However, the feature selection of these methods relies heavily on artificial markers, resulting in unsatisfactory classification accuracy.

In BCI studies, deep learning methods have attracted the attention of researchers in medical applications [8], and studies have found that deep learning methods perform higher accuracy in MI decoding tasks than traditional machine learning methods [9]. The convolutional neural networks (CNN) have been commonly used for automatic feature extraction [10] and classification [11]. Schirrmeister et al. proposed the DeepConvNet and ShallowConvNet models based on the FBCSP and CNNs for feature extraction, which improved the four-class average accuracy by 7% on the BCIC public motion imagination dataset [12]. Lawhern et al. proposed the EEGNet algorithm for feature extraction and integration, which contains the depth-wise separable CNNs. The number of parameters is much less than DeepConvNet and ShallowConvNet, and the model reached fine classification accuracy in MI [13]. Izzuddin et al. proposed a more compact MI decoding architecture based on EEGNet, which use a parameterized SincNet layer for bandpass filtering in the first layer CNN [14]. The deep learning methods based on CNN for EEG classification is a promising decoding technological path. There is a problem that the input type of CNN networks is limited to European data, which ignores the characteristics of the non-Euclidean space of the brain and cannot fully extract the spatial features of the MI signals.

Recently, models based on graph structures have been proposed to solve the problem of feature extraction in non-Euclidean space [15]. Scarselli et al. proposed a Graph Neural Network (GNN) model suitable for graph structures and nodes, and designed a function that maps the graph and its nodes to a higher-dimensional Euclidean space [16]. This paper laid the foundation for the subsequent research of GNN. Kipf et al. proposed the Graph Convolutional Network (GCN) through the localized first-order approximation of spectral graph, which is an extension of GNN [17]. At present, the graph convolution network has rarely been applied to the MI task. Zhang et al. constructed three kinds of distance brain views according to the spatial location of EEG electrodes, and performed a graph embedding of MI signals to learn spatial and temporal features from the time periods with the highest discrimination [18]. Sun et al. proposed an Adaptive Spatiotemporal Graph Convolutional Network (ASTGCN), which dynamically integrated electrode channel information by constructing an adaptive graph convolutional layer to realize the classification of the MI tasks [19]. Hou et al. proposed a MI classification method based on graph convolutional networks and constructed a Laplacian graph representing the electrode topological relationship based on the absolute Pearson matrix of EEG signals [20]. However, these methods only focus on a single aspect of the physical location connection, and ignore the importance of the functional connection between brain regions.

In addition, the attention mechanism has recently made great progress in the fields of computer translation [21] and vision [22], which can dynamically assign weights to input vectors for feature selection. Researchers have tried to apply attention mechanism to motor imagery decoding work, Li et al. proposed a CNN model based on the attention mechanism (MS-AMF), which extracted spatiotemporal features at multiple scales based on the Squeeze-and-Excitation (SE) channel attention method [23]. Zhang et al. reported an automatic channel selection (ACS) strategy based on the SE method for automatically assigning channel weights in EEG signals [24]. Liu et al. developed a distinguishable spatial–spectral feature learning method based on FBCSP for preliminary feature extraction and two SE blocks for feature recalibration [25]. Yu et al. proposed a Lightweight Feature Fusion method based on the improved convolutional block attention module (CBAM) for feature selection operations [26]. However, these methods focus on the attention information between local channels and have limitations in sensing the overall dependence of EEG signals. The self-attention method has been applied to emphasize the global expression capability of discriminable EEG features in emotion recognition [27] and sleep stage classification [28], but it has not been leveraged currently for the MI graph representation classification task.

To overcome these above limitations, we propose a multi-view graph convolutional attention network (MGCANet) algorithm for decoding MI-EEG signals. Specifically, we design a multi-view graph convolutional network with a residual structure and a temporal convolutional network for sufficient feature extraction. Subsequently, we employ the multi-head self-attention for feature selection.

The major contributions of our study are summarized as follows:

(1) We constructed different representation of brain views based on physical distance and functional connection, which can sufficiently express the topological relationship of brain regions in MI signals for subsequent spatial feature extraction.
(2) We designed a residual graph convolutional network called ResChebyNet by combing the advantage of the residual learning and Chebyshev functions. In order to avoid the gradient vanishing problem caused by the increase of the layers of the graph convolutional network.
(3) We developed an adaptive-weighted fusion (Awf) module for collaborative integration of features extracted from different brain views, which can enhance the reliability and accuracy of feature fusion.
(4) We introduced the multi-head self-attention method in the classification framework, which can extend the receptive field of MI signals to a global scale and effectively enhance the expression ability of important features to improve decoding accuracy.

The rest of this paper is as follows. Section 2 contains the details of the proposed approach. Section 3 describes the results of the experiments. Section 4 contains discussion of the proposed approach. The conclusion is in Section 5.

## 2. Methods

### 2.1. Overall Architecture

**Figure 1:**
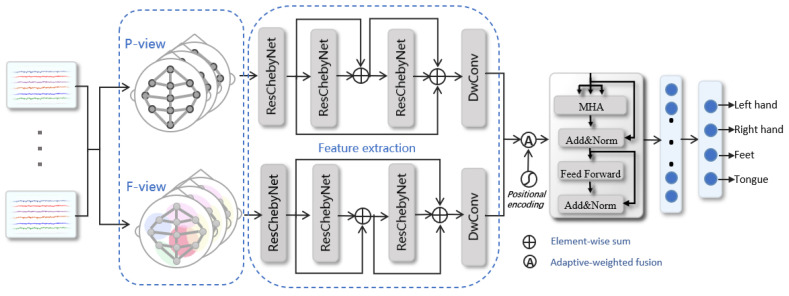
The structure of the MGCANet method. P-view and F-view represent the physical-distance-based brain view and functional-connectivity-based brain view, respectively. DwConv and MHA stand for the depth-wise convolutional layer and multi-head attention layer.

The overall architecture of the proposed MGCANet model is illustrated in Figure 1. It contains four components: multi-view on brain network, spatial–temporal feature extraction, feature selection, and classification. First, multiple views of the brain connection are designed for fully reflect the topological relationships among brain regions, then the ResChebyNet is applied for extracting spatial features and the depth-wise convolutional layer is employed for extracting temporal features. The Awf method is developed to combine the features obtained from different views adaptively. After that, the self-attention mechanism is utilized for feature selection. Finally, the obtained features are input into the classifier for classification.

### 2.2. Multi-View on Brain Graph

As neuroscience research suggests that, during MI tasks, there will be interactions among the functional regions of the brain. Existing MI modeling methods are limited to focus on a single aspect of physical spatial connections. In this case, we construct physical-distance-based brain view and functional-connectivity-based brain view, respectively. Different views reflect different spatial correlations, which integrate the two aspects of correlation to enhance the spatial information expression of MI-EEG signals.

In this paper, the original MI-EEG signal sequence is defined as $Χ=[x_{1},\ldots ,x_{N}]\in R^{N\times T_{\mathrm{time}}}$, where N represents the number of electrodes for EEG acquisition, $T_{\mathrm{time}}$ represents the number of time sampling points, and $x_{i\;\in \left[1,N\right]}\in R^{T_{\mathrm{time}}}$ represents the one-dimensional EEG signal. The MI brain network is defined as graph G=(V,E,A), where V represents the set of vertices, each vertex represents an electrode of EEG, which represents the number of vertices in the MI brain network, that is, |V|=N, E represents the set of edges, i.e., the connections between EEG electrodes, and A represents the adjacency matrix of the motor imagery brain network G.

**Figure 2:**
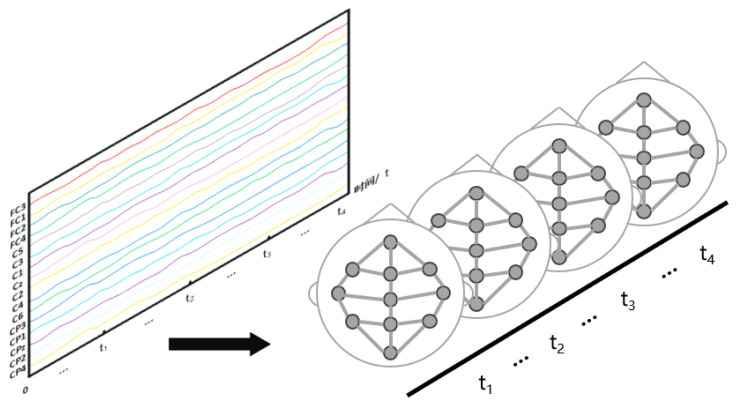
Graph conversion of EEG signals.

As shown in Figure 2, each time node of the EEG signal $X\in R^{N\times T_{\mathrm{time}}}$ is regarded as a graph, and multiple brain graphs are constructed according to the number of time nodes. We construct the physical-distance-based brain view $G^{P}$ and the functional-connectivity-based brain view $G^{F}$ from the brain graph, respectively. They are described separately below.

#### 2.2.1. Physical-Distance-Based Brain View

**Figure 3:**
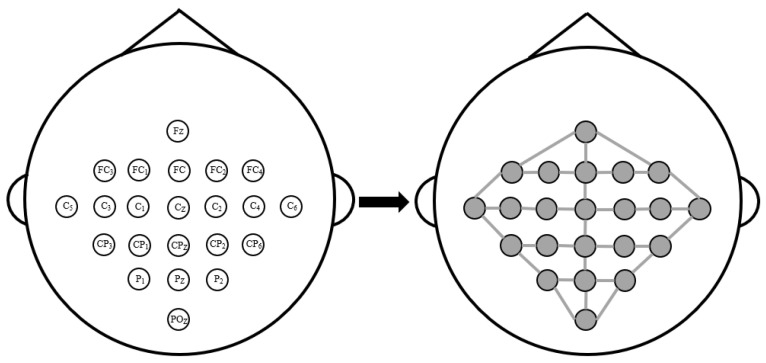
The diagram of the constructed adjacency matrix based on physical distance.

We construct the adjacency matrix based on the natural spatial distribution of EEG electrodes and quantify the degree of connection between different electrodes by the physical distance, the diagram is illustrated in Figure 3. Research has shown that the closer the EEG channels are physically located, the greater the interaction between them, while the interaction between distant channels is smaller. Inspired by the D-Graph [18], we use the distance-based connection method to calculate the distance among electrode nodes, thereby reflecting the connection between physical spatial locations and enhancing the representation ability of brain region information.

We define the set of the distance between any two EEG nodes as $D_{\mathrm{dis}}=\{d_{\mathrm{ij}}|\left(p^{i},p^{j}\right)\in V^{2},i\neq j\}$, where V is the set of all nodes, $p^{i}$ and $p^{j}$ are two different electrode nodes in V, $d_{\mathrm{ij}}=\sqrt[{2}]{{\left(x_{i}-x_{j}\right)}^{2}+{\left(y_{i}-y_{j}\right)}^{2}+{\left(z_{i}-z_{j}\right)}^{2}}$ is the Euclidean distance between node $p^{i}$, and $p^{j}$, $x_{I}$, $y_{i}$, and $z_{i}$ are the physical coordinates of the nodes. We regard the two nodes are adjacent when the distance between two nodes is less than the average value of $D_{\mathrm{dis}}$, and the distance between two nodes is defined as the average distance of other adjacent nodes to this node. The diagram of the constructed adjacency matrix based on physical distance is shown in Figure 3 (taking the BCIC IV 2a dataset as an example). The adjacency matrix $A_{\mathrm{distance}}$ of the physical-distance-based brain view is expressed as follows:

(1) $$A_{\mathrm{distance}}=\left\{\begin{array}{c} \frac{1}{d_{\mathrm{ij}}}\;if\;d_{\mathrm{ij}}<M\left(D_{\mathrm{dis}}\right)\\ 0\;if\;{\;d}_{\mathrm{ij}}\geq M\left(D_{\mathrm{dis}}\right)\\ \frac{1}{M(\{d_{\mathrm{iq}}|d_{\mathrm{iq}}<M(D_{\mathrm{dis}}),q\in \left[1,N]\}\right)}\;if\;i=j \end{array}\right.$$

where the $M\left(D_{\mathrm{dis}}\right)$ is the average function of distance set $D_{\mathrm{dis}}$.

#### 2.2.2. Functional-Connectivity-Based Brain View

During the task of the motor imagery, there will have collaborative neural activity between different brain regions, and generate functional spatial topological relationships. In this case, we construct the brain view based on the functional connections to dynamically learn the functional graph of MI-EEG. We adaptively construct adjacency matrices based on the connection relationship between different channels to further enhance the expression of spatial correlation information.

Specifically, different EEG channels are regarded as nodes in the graph network, and the matrix function $A_{function}$ is defined to represent the functional connection relationship between node p and node q, which is expressed as:

(2) $$A_{function}=\frac{\exp\left(\mathrm{ReLU}\left(\omega^{T}\left|x_{p}-x_{q}\right|\right)\right)}{\sum_{q=1}^{N}\exp\left(ReLU\left(\omega^{T}\left|x_{p}-x_{q}\right|\right)\right)}$$

where $x_{i\;\in \left[1,N\right]}\in R^{T_{\mathrm{time}}}$ is the input of the EEG signal, $\omega ={\left[\omega_{1},\omega_{2},...,\omega_{T_{\mathrm{time}}}\right]}^{T}\in R^{T_{\mathrm{time}}}$ is a learnable parameter, and ReLU is the activation function to ensures that $A_{function}$ is a non-negative function. The Softmax function is used to normalize the rows of the matrix function. The learnable parameter ω is updated by minimizing the loss function, which is expressed as:

(3) $$L_{\mathrm{function}}=\sum_{p,q=1}^{N}{{\left|\right|x}_{p}-x_{q}\left|\right|}_{2}^{2}A_{function}+\lambda {\left|\right|A_{function}\left|\right|}_{2}^{2}$$

where ${\left|\right|x}_{p}-x_{q}|$| represent the functional distance between the two nodes. The regularization parameter λ is used to reduce the sparsity of the matrix and avoid generating trivial solutions. We adopt the cross-entropy loss function as the supervised classification loss, which is defined as:

(4) $$L_{crossloss}=-\frac{1}{N}\sum_{k=1}^{K}\sum_{n=1}^{N}y_{n}\log\left({\hat{y}}_{n}\right)$$

where N represents the number of samples, K represents the number of categories, $y_{n}$ represents the sample label, and ${\hat{y}}_{n}$ represents the probability of identifying the sample label as category k. The functional loss $L_{function}$ is adopted as a regularization term to the loss function to form the final loss function. The total loss function $L_{loss}$ of the model can be expressed as follows:

(5) $$L_{loss}=L_{crossloss}+L_{function}$$

In order to achieve adaptive learning of graph structure information and improve the model’s ability to process graph data, we add the trainable parameter matrix W to the adjacency matrix $A_{distance}$ and $A_{distance}$ before the graph convolutional layer for adaptive adjustment:

(6) $$\left\{\begin{array}{c} H_{\mathrm{distance}}=(A_{distance}⨀W_{1})\\ H_{\mathrm{function}}=(A_{function}⨀W_{2}) \end{array}\right.$$

where $A_{distance}$ and $A_{function}$ represent the physical-distance-based adjacency matrix and the functional-connectivity-based adjacency matrix, respectively. $W_{1}\in R^{N\times N}$ and $W_{2}\in R^{N\times N}$ are learnable parameter matrices and $H_{\mathrm{distance}}$ and $H_{\mathrm{function}}$ are the output adjacency matrix after adaptive adjustment.

### 2.3. Spatial–Temporal Feature Extraction

#### 2.3.1. Spatial Feature Extraction

Graph convolution based on spectral graph theory can be used to extract spatial feature in the spatial dimension. We develop a method based on the Chebyshev graph convolution [17] to extract the spatial feature of the MI signals. The Chebyshev graph convolution approach is reviewed in this section.

For graph G, its corresponding Laplacian matrix $L\in R^{N\times N}$ can be expressed as:

(7) L=D−A

where the degree matrix $D\in R^{N\times N}$ is the diagonal matrix consisting of the degrees of graph nodes, $D_{ii}=\sum_{j}A_{i,j}$, A is the adjacency matrix. Since ***L*** is a real symmetric matrix, it can be normalized and expressed as:

(8) $$L=I_{N}-D^{-\frac{1}{2}}AD^{-\frac{1}{2}}=U{\Lambda U}^{T}$$

where $I_{N}$ is the identity matrix, Λ is the eigenvalue matrix of L, and U is the orthonormal matrix composed of the eigenvectors of matrix L. Assume the input of the graph is x, the graph Fourier transform is denoted as:

(9) $$\hat{x}=U^{T}x$$

The inverse graph Fourier transform is expressed as:

(10) $$x=U\hat{x}$$

The graph convolution with input data x and filter $g_{\theta }$ can be expressed as:

(11) $$x*g_{\theta }=U\left(U^{T}g_{\theta }⨀U^{T}x\right)=U\hat{g_{\theta }}U^{T}x$$

where ⨀ represents the Hadamard product and $\hat{g_{\theta }}=U^{T}g_{\theta }$. Let $g_{\theta }$ be the function $g_{\theta }(\Lambda )$ of the eigenvalue matrix of L.

Since the operation has high computational complexity and lack of locality, Defferrard et al. [17] introduced the K-order truncated expansion of Chebyshev polynomials to approximate the filtering operation as follows:

(12) $$g_{\theta }=g_{\theta }(\Lambda )\approx \sum_{k=0}^{K}\theta_{k}T_{k}\left(\tilde{\Lambda }\right)$$

(13) $$\tilde{\Lambda }=\frac{2}{\lambda_{max}}\Lambda -I_{N}$$

where $\lambda_{max}$ is the largest eigenvalue of L, θ is the coefficient of Chebyshev polynomial. The Chebyshev graph convolution (ChebyNet) with input data x and filter $g_{\theta }$ can be expressed as:

(14) $$x*g_{\theta }\approx \sum_{k=0}^{K}\theta_{k}T_{k}\left(\tilde{L}\right)x$$

(15) $$\tilde{L}=\frac{2}{\lambda_{max}}L-I_{N}$$

where $T_{k}$ is the Chebyshev polynomials which is recursively defined as:

(16) $$\;\left\{\begin{array}{c} T_{0}\left(\tilde{L}\right)=1\;\;\;\;\;\;\;\;\;\;\;\;\;\;\;\;\;\;\;\;\;\;\;\;\;\;\;\;\\ T_{1}\left(\tilde{L}\right)=\tilde{L}\;\;\;\;\;\;\;\;\;\;\;\;\;\;\;\;\;\;\;\;\;\;\;\;\;\;\;\;\\ T_{k}\left(\tilde{L}\right)=2\tilde{L}T_{k-1}\left(\tilde{L}\right)-T_{k-2}\left(\tilde{L}\right),\;\;\;k>1 \end{array}\right.$$

**Figure 4:**
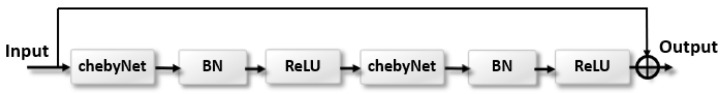
The structure of the ResChebyNet.

Since deep graph convolutional network can aggregate high-order features, we build the ReschebyNet block combining the ChebyNet and residual learning [29] to alleviate the gradient vanishing problem caused by multi-layer graph convolutional layers, the structure of ReschebyNet block is shown in Figure 4. A ChebyNet layer contains the graph convolutional layer, the normalization layer and the activation layer. We utilize the ChebyNet as the graph convolutional layer for feature extraction. This is closely followed by a batch normalization layer [30] and an activation function ReLU, which is used to accelerate network convergence speed and perform nonlinear calculations. We build the ResChebyNet block with two sequential ChebyNet layers in inter-block residual connection and add the input of the block directly to the output of the block in skip connection.

The over-smoothing phenomenon will occur when the scale of the graph convolutional network aggregation is gradually expanded to all nodes of the graph, which will cause the gradient vanishing in backpropagation. To overcome the defect of deep GCN, we use the internetwork-block residual structure for the connection between each ResChebyNet block, and the local structure of the graph obtained by the convolutional network of the previous block of graph is used as the input to the next block, as shown in Figure 1. During the process of spatial feature extraction, the sum of the input of each block and the output of the last block is taken as the final extracted spatial feature.

#### 2.3.2. Temporal Feature Extraction

After extracting spatial feature through the proposed ResChebyNet, we use a depth-wise convolutional layer with a convolution kernel size of (1, 25) to extract temporal features in the temporal dimension. A batch normalization layer and an activation layer with ELU function is performed after the depth-wise convolutional layer, which is employed to speed up training. Then, an average pooling layer with kernel size of (1, 4) is utilized to compress temporal features and integrate the information.

### 2.4. Adaptive-Weighted Fusion

Existing research [19,31] generally uses element-wise addition or concatenate operation to directly concatenate the features extracted from different graph convolution branches, which ignore the potential for collaboration of both. Therefore, we propose an adaptive-weighted fusion (Awf) module, which utilizes the complementary advantages of features from two different views to maximize the representativeness of each feature and improve the decoding performance. 

**Figure 5:**
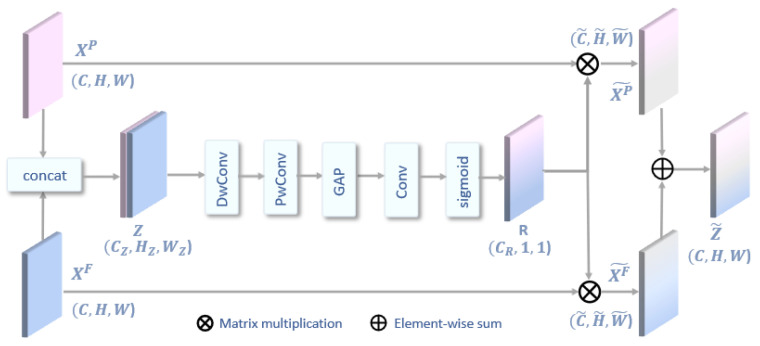
The structure of the adaptive-weighted fusion module. DwConv and PwConv denote the depth-wise convolutional layer and the pointwise convolutional layer and GAP denotes the global average pooling layer.

As shown in Figure 5, $X^{P}$ and $X^{F}$ represent the features from the physical-distance-based brain view and functional-connectivity-based brain view, respectively. First, a concatenation operation is used for connect the two feature sets to obtain the fused feature Z. A depth-wise convolutional layer with kernel size 3 and a pointwise convolutional layer with kernel size 1 is used to reduce channel dimension. A global average pooling (GAP) layer is used to reduce the model parameters and computation. Then, a convolutional layer with kernel size 1 without bias is used to integrate the channel information, and a sigmoid function is used to calculate the correlation coefficient matrix *R*. The correlation coefficient matrix R can be expressed as:

(17) $$R=\sigma (f_{Conv}(GAP(f_{PwConv}\left(f_{DwConv}\left(Z\right)))\right))$$

where σ is the sigmoid function, $f_{conv}$ denotes convolutional operation, and GAP represents global average pooling operation. Then, the coefficient matrix is multiplied with the input feature to obtain $\tilde{X^{P}}$ and $\tilde{X^{F}}$, which are added together to get the final output feature $\tilde{Z}$. The output of the Awf module can be expressed as:

(18) $$\tilde{Z}=ReLU(\tilde{X^{P}}+\tilde{X^{F}})$$

where ReLU is the activation function.

### 2.5. Self-Attention Feature Selection

Each convolution operation has a limited receptive field, which may lead to the loss of global feature information during EEG feature extraction and affect the classification accuracy of the model. In order to solve the problem of limited receptive field size caused by the convolutional structure, the self-attention mechanism is introduced to capture the global dependencies of EEG signals. We combine the multi-head attention (MHA) [32] method in MGCANet to calculate the attention strength between nodes, and assign more attention weights to the features that have a higher contribution to the classification. Simultaneously, multiple attention modules are used to learn the global dependence of MI-EEG signals from different perspectives to represent more feature information.

The input of MHA method is denoted as $\tilde{Z}$, which are obtained from the prior blocks. First, the input features are transformed into query vectors (*Q*), key vectors (*K*), and value vectors (*V*) with the same shape by multiplying three corresponding weight matrices. Second, a dot product operation is performed on each *Q* and all remaining *K* to compute the degree of correlation between vectors. The self-attention weight is calculated by matrix multiplication and SoftMax function, which can express as follows:

(19) $$Attention\left(\tilde{Z}\right)=softmax\left({QK}^{T}/\sqrt{d_{k}}\right)V$$

where $\sqrt{d_{k}}$ is a scaling factor to ensure the stability of gradient. The self-attention feature vector can be obtained by multiplying the self-attention weight and perform element-wise summation with the value vector. Multi-head attention concatenates multiple self-attention layers to obtain internal features of different representation subspaces and achieving richer feature information representation. MHA is expressed as follows:

(20) $$MHA\left({\tilde{Z}}_{Q},{\tilde{Z}}_{K},{\tilde{Z}}_{V}\right)=\left[{head}_{0};\ldots ;{head}_{h-1}\right]W^{O}$$

(21) $${head}_{i}=Attention\left({\tilde{Z}}_{Q}W_{i}^{Q},{\tilde{Z}}_{K}W_{i}^{K},{\tilde{Z}}_{V}W_{i}^{V}\right)$$

where $W_{i}^{Q}$, $W_{i}^{K}$, and $W_{i}^{V}$ represent the weight matrices for each group of *Q*, *K*, and *V*, respectively, ${head}_{i}$ represents the representation subspaces of ith attention head, and MHA denotes merging all groups of attention heads together to obtain the final output that contains all the attention head information. Layer normalization is added after the MHA layer to normalize the representation. A feed-forward (FF) block contains two fully connected layers is applied to improve calculation efficiency.

### 2.6. Classification

After feature extraction, the spatio-temporal feature maps obtained by prior blocks are concatenated along the channel axis and compress them into a one-dimensional feature vector. Finally, two fully connected layers and a SoftMax function was performed as the classifier to recognize the target limbs.

## 3. Experiments and Results

We conducted several experiments on two open datasets and compared the performance of the proposed MGCANet with other typical MI classification methods. The details of experimental datasets, setup, and results are shown as follows.

### 3.1. Dataset

Two frequently used public MI datasets, BCI Competition IV 2a dataset [33] and OpenBMI dataset [34], are utilized for the experiments.

#### 3.1.1. BCI Competition IV 2a Dataset

BCI Competition IV 2a dataset (denoted as BCIC IV 2a below) is a public dataset containing the motor imagery records of nine subjects recorded on two different days. It has two sessions including four types of motor imagination tasks (left hand, right hand, feet, and tongue). Each session includes a total of 288 trials, with 72 trials in each category. EEG signals are collected by using 22 electrodes and sampled at a sampling rate of 250 Hz. We intercept the data from 0 to 4 s after the prompt as a trial, and each trial is a matrix with a dimension of channels (22) × sampling points (1000).

#### 3.1.2. OpenBMI Dataset

The OpenBMI dataset is a public dataset containing the motor imagery records of 54 subjects recorded the tasks of left hand and right hand. It has two sessions, where each session includes a total of 200 trials, with 100 trials in each category. EEG signals are collected by using 62 electrodes and sampled at a sampling rate of 1000 Hz. According to the settings of the original dataset, the raw data are downsampled to 250 Hz and 20 electrodes from the motor cortex region are selected for analysis, namely, FC5, FC3, FC1, FC2, FC4, FC6, C5, C3, C1, Cz, C2, C4, C6, CP5, CP3, CP1, CPz, CP2, CP4, and CP6. We intercept the data from 0 to 4 s after the prompt as a trial, and each trial is a matrix with a dimension of channels (20) × sampling points (1000).

### 3.2. Experimental Setup

The experiments were implemented with the PyTorch library on a workstation with i9-13900K CPU and RTX4090 GPU. Scikit-learn was utilized to calculate the confusion matrix.

For the preprocessing, we utilized the first-order Butterworth filter to filter the EEG signals and performed exponential moving average normalization on the filtered signal [35] to reduce the impact of numerical differences. We performed cross-session evaluation experiment on BCIC IV 2a and OpenBMI. The signals in session 1 were divided into training set, and the signals in session 2 were divided into evaluation set. In the training set, 80% of the data was randomly selected as the training set and the remaining 20% as the validation set. For the training settings, the batch size was set as 64, and max number of epochs was 400. The adaptive moment estimation (Adam) optimizer [36] was used for model optimization with a learning rate set as 0.001. Wilcoxon Signed-Rank Test was utilized to evaluate the statistical significance.

### 3.3. Compared Methods

We compared five representative methods published in recent years. The description of compared methods are given as follows.

- DeepConvNet [12]: DeepConvNet is a general-purpose architecture which combines temporal convolution and spatial convolution operations. It consists of five convolutional layers. We trained this model in the same way as the original paper.
- EEGNet [13]: EEGNet designs a lightweight CNN for EEG decoding. The method was modified according to Borra et al. [37], and is suitable for 128 Hz EEG signals (as opposed to 250 Hz signals in our study).
- Sinc-ShallowNet [37]: This method extracts features of EEG signals by stacking temporal sinc-convolutional layers and spatial convolutional layers. We reproduced the author’s design and obtained comparable performance.
- G-CRAM [18]: G-CRAM constructs three graph structures through the positioning information of EEG nodes and use a convolutional recurrent model for feature extraction. We adjusted the input format based on the original paper.
- BiLSTM-GCN [38]: BiLSTM-GCN uses the BiLSTM with the attention model to extract features and uses the GCN model based on Pearson’s matrix for feature learning. We performed corresponding reproduction operations according to the design of the original paper.
- EEG-Conformer [39]: The method consists of three parts: a convolution block, a self-attention block, and a fully connected classifier. We performed corresponding reproduction operations according to the published code in the paper.

### 3.4. Results

#### 3.4.1. Comparison Experiments

We compared the performance of our model with the performance of five representative methods on the MI datasets. The results of the cross-session experiments are listed in Table 1. We presented the average accuracy and kappa value as performance metrics, where the kappa value is used to measure the stability of the model. The standard deviation denotes as std, and the highest accuracy (acc) and kappa value are shown in bold. It can be found that the average acc and kappa value of the MGCANet method are significantly higher than the compared methods (*p*-value < 0.05), and the standard deviation is the lowest. This indicates that the proposed method has superior classification performance and better robustness. The MGCANet model reaching an average classification accuracy of 78.26% on BCIC IV 2a, which is 2.89% higher than the compared EEG-Conformer method. And the MGCANet model reaching an average classification accuracy of 73.68% on OpenBMI, which is 5.04% higher than Sinc-ShallowNet method in two-class scenario.

DeepConvNet methods directly use the spatio-temporal matrix of EEG signal without fully considering the topological relationship between EEG electrodes. Therefore, the average classification of the DeepConvNet methods is 11.39% lower than the proposed model. The EEGNet and the sinc-ShallowNet methods contains multi-layer CNNs for feature extraction. The average accuracy of the two methods is unsatisfied due to the lack of consideration of the topological connection of brain regions. The G-CRAM method only focuses on the physical position of the electrodes, ignoring the influence of the functional connection of the brain regions. For the BiLSTM-GCN method, the ability to extract global information is relatively weak, which results in lower classification performance than the proposed method. EEG-Conformer method uses the self-attention method to learn global temporal dependencies of EEG features, which has lower mean accuracy due to the lack of analysis of the topological connection among brain regions.

#### 3.4.2. Confusion Matrix

**Figure 6:**
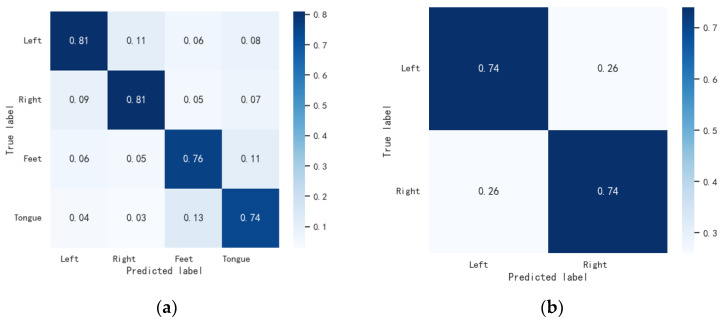
The confusion matrix of MGCANet. (**a**) The confusion matrix on BCIC IV 2a. (**b**) The confusion matrix on OpenBMI.

We plotted the confusion matrix of the proposed method for classification on two datasets to evaluate the performance of the algorithm. The confusion matrix is shown in Figure 6. The horizontal axis of the matrix stands for the predicted result labels of the samples, while the vertical axis stands for the actual labels. Among them, the percentage in each column of the matrix identify the proportion of the number of samples to the class. From Figure 6, it can be seen that the proposed method has a higher recall rate in the left-hand and right-hand categories than the other two categories on the BCIC IV 2a dataset. This is because the spatial features induced by left-hand and right-hand motor imagery are more discriminable, indicating that the proposed method can effectively distinguish categories with significant differences. The misclassifications are frequently occurred between the left-hand and right-hand MI. Moreover, the feet MI tasks are frequently misclassified as tongue tasks. This is due to the complexity of the subjects’ thinking activities, and body categories with small inter-category differences are easily confused. On the OpenBMI dataset, the proposed method can correctly classify the vast majority of the left-hand and right-hand categories, and the two types of samples have similar recall rates.

#### 3.4.3. Ablation Study

The ablation study was performed on two datasets to verify the necessity of each component in MGCANet model, and the result is shown in Table 2.

(a) MGCANet without using the physical-distance-based brain view (denoted as ‘w/o P-view’);

(b) MGCANet without using the functional connection-based brain view (denoted as ‘w/o F-view’);

(c) MGCANet without using the Residual structure (denoted as ‘w/o Res’);

(d) MGCANet without using the adaptive-weighted fusion (denoted as ‘w/o Awf’); 

(e) MGCANet without using the multi-head attention method (denoted as ‘w/o MHA’);

(f) Complete MGCANet method.

Table 2 shows the result that the average accuracy of MGCANet method is significantly higher than compared methods on two datasets. This demonstrates that each contribution proposed is effective and necessary. On the BCIC IV 2a, the accuracy of experiments (a) and (b) decreased significantly by 3.43% and 3.59%, respectively, indicating that the P-view and F-view have a greater effect on the improvement of model decoding performance. On the OpenBMI dataset, the accuracy of experiments (a) and (c) decreased significantly by 3.73% and 3.96%, respectively, indicating that the P-view and residual structure played a significant role in improving the decoding performance of the model. This also indicates that the emphasis of network models varies across different datasets.

#### 3.4.4. Visualization

**Figure 7:**
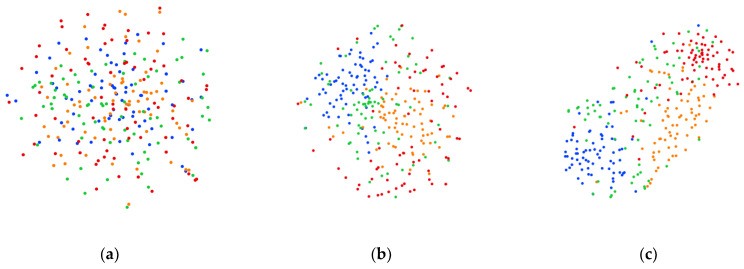
Visualization results on BCIC IV 2a dataset (**a**) Data distribution of initial data. (**b**) Data distribution after the feature extraction block. (**c**) Data distribution after the feature selection block.

**Figure 8:**
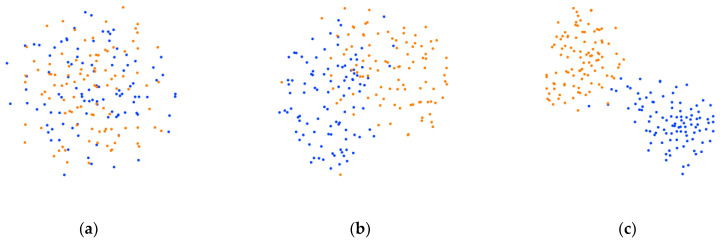
Visualization results on OpenBMI dataset (**a**) Data distribution of initial data. (**b**) Data distribution after the feature extraction block. (**c**) Data distribution after the feature selection block.

We used the t-SNE method [40] to visualize the feature distribution on the two datasets. The quality of the proposed model was analyzed from the visualization results, as shown in Figure 7 and Figure 8. Yellow, blue, green, and red represent the left hand, right hand, feet, and tongue MI tasks, respectively. In each figure, Figure 7a–c and Figure 8a–c represent the data distribution of initial data, data distribution after the feature extraction block, and data distribution after the feature selection block, respectively. It can be seen that the distribution of the initial data is difficult to discriminate. Each category of MI data shows clustering distribution in two-dimensional space after the feature extraction block. After being processed by feature selection block, the distance between samples of different categories becomes longer and each category of data shows obvious clustering distribution characteristics. This indicates that each block in the proposed method exhibits relatively good discriminability.

## 4. Discussion

In this study, the proposed MGCANet model improved the accuracy by fusing the topological association of brain regions and global attention dependency of EEG signal sequence. First, we consider the physical and functional associations of brain regions to construct different views to achieve information complementarity and emphasize the spatial feature expression of MI signals. Secondly, we use the residual learning to avoid the gradient vanishing and over-smoothing problems caused by increasing GCN depth, which can learn high-order features and make the model easily converged. Thirdly, we introduce the multi-head attention for capturing global feature information, which enable the model to obtain more abundant features. Besides, we proposed a method to adaptively fusion the features for building a more efficient model. The effectiveness of the proposed model is demonstrated through the comparison experiments and the ablation study in Section 3.4, which can realize higher precision of MI classification.

### 4.1. Visualization of the Brain Topographical Map

**Figure 9:**
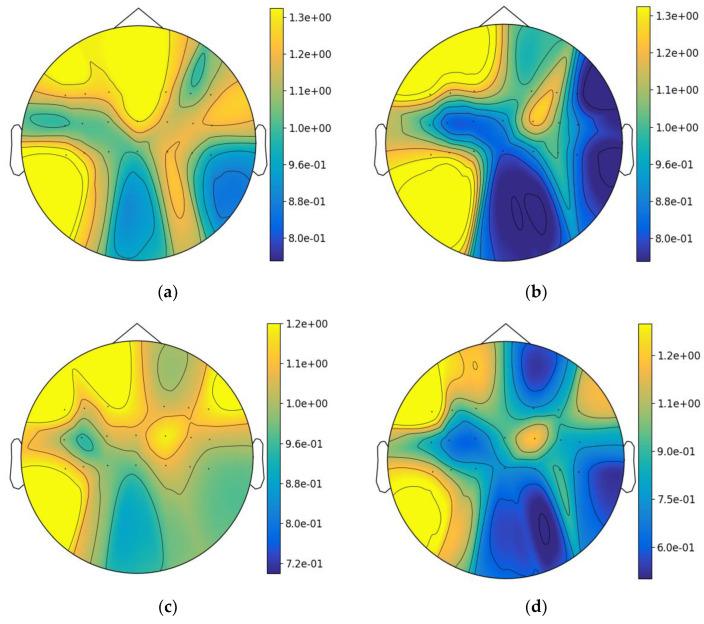
Topographical distribution of spatial features in OpenBMI: (**a**) the topography of original samples of subject 17; (**b**) the topography of enhanced samples by the functional connection of subject 17; (**c**) the topography of original samples of subject 48; (**d**) the topography of enhanced samples by the functional connection of subject 48.

To explore the impact of the functional connection-based brain view on spatial feature distribution, we performed brain topographical visualization on the OpenBMI dataset. As shown in Figure 9, Figure 9a,b represent the left-hand motor imagery samples of subject 17, and Figure 9c,d represent the left-hand motor imagery samples of subject 48. It can be seen that there are differences in event-related desynchronization and event-related synchronization (ERD/ERS) when different subjects (subject 17 and subject 48) perform the same motor imagery task (left hand). The specific performance is that the activation location and intensity are different, which shows that there are obvious individual differences in the MI-EEG signals between different subjects. In addition, it can be seen that the energy distribution of the left-hand motor imagery samples after the enhancement of spatial features changes more significantly than that of the original samples, and both can reflect the energy spatial distribution of left-hand motor imagery.

### 4.2. Selection of Parameters

In this study, we adjusted the parameters of the proposed method to obtain better classification results. The K-order of the Chebyshev polynomials, the number of graph convolutional layers, the number of attention heads, and the maximum norm of weights in fully connected layers have the influence on the classification performance. The range of values was determined according to commonly used values in deep learning field. The parameter selection experiment started from K-order, and then adjusted the number of the graph convolutional layers, the number of attention heads, and the maximum norm sequentially. We kept the values of other parameters unchanged when tuning each parameter. The result of different parameters selection is shown in Table 3, and the highest average classification accuracy is bolded.

As can be seen from Table 3, the optimal performance can be obtained with K = 3, the number of layers is set as 3, the number of attention heads is set as 8, and maximum norm is set as 0.5. The network with K value set as 3 has higher classification accuracy than the network with K value set as 1 or 2. This shows that the larger value of K-order in the Chebyshev polynomial is, the larger the receptive radius of the convolutional kernel is. Therefore, the node can capture more feature information from other nodes for aggregation. However, the classification accuracy of the network with K value set as 4 is lower than that of the network with K value set as 3, which indicates that when the sensory radius of the convolutional kernel increases, the node will add more irrelevant information while capturing more feature information. In addition, it can be observed that the number of graph convolutional layers also affects the accuracy of model classification. Increasing the number of graph convolutional layers can increase the expressiveness of the model, but at the same time, it can increase the complexity of the model. The classification accuracy of the four-layer graph convolutional network is lower than that of the three-layer graph convolutional network, which indicates that increasing the number of graph convolutional layers may lead to overfitting. The classification accuracy continues to increase when the number of attention heads increases. This indicates that increasing the number of attention heads sever the purpose to obtain more comprehensive features and avoiding the model from relying on certain features for decoding. However, the increase of the number of attention heads will increase the parameters, resulting in overfitting and affecting the improvement of accuracy.

### 4.3. Ablation Study of the Adaptive-Weighted Fusion

To verify the effectiveness of the proposed adaptive-weighted fusion method, we compare it with the other two feature fusion methods, the element-wise addition (denote as add) and concatenation (denote as concat). The experimental result is shown in Table 4. It can be observed that on the BCIC IV 2a dataset, the average accuracy of the proposed fusion method is 1.78% and 1.01% higher than that of the comparison method, respectively. For the OpenBMI dataset, the average accuracy of the proposed fusion method is improved by 1.44% and 0.83%, respectively, compared with the comparison methods. The experimental results further demonstrate the effectiveness of the proposed Awf method.

### 4.4. The Influence of Different Attention Methods

In this work, we introduced the multi-head attention (MHA) method for feature selection by capturing global attention information, which effectively integrates local and global features. We evaluate the classification performance of four different attention methods on two datasets, namely Squeeze-and-Excitation (SE) [41], Efficient Channel Attention (ECA) [42], and Shuffle Attention (SA) [43], and the result is shown in Table 5. The standard deviation denotes as std, and the highest accuracy (acc) is shown in bold. It can be seen that the classification accuracy of the MGCANet model combined with MHA is higher than that of the model combined with other attention blocks. The model combined with SE allocates attention weights based on the information between feature maps, only considering the attention in the channel dimension and ignoring the capture of attention in the spatial dimension. Since the SA method sets certain feature map groups, it will reduce the effect of channel attention and affect the classification accuracy of the model combined with SA. The model combined with MHA can calculate the feature correlation in the spatial–temporal dimensions and global attention information, which can achieve effective attention weight allocation and improve the decoding performance.

Although the proposed model has achieved good results on the MI decoding tasks, the number of parameters still needs further reducing to build a more lightweight classification model. Future work intends to further explore the ways to optimize the network structure from the perspective of reducing the number of parameters and reducing the number of attention blocks.

## 5. Conclusions

In this paper, we propose a multi-view graph convolutional attention network (MGCANet) model for motor imagery classification. According to the topological relationship of brain regions during MI tasks, different brain views are constructed based on physical and functional connection to enrich the representation of spatial association information. Considering that the increase in the number of GCN layers can cause gradient vanishing and network degradation problems, we construct the ResChebyNet through residual learning structure, and design an adaptive-weighted fusion strategy to fuse the features from different brain graphs to improve the ability of feature learning. Besides, we introduced the multi-head self-attention method to learn global dependencies and further improve the classification accuracy of the model. The experimental results on public datasets demonstrate the efficiency of the proposed MGCANet and can improve the performance of MI decoding tasks.

## Figures and Tables

**Table 1:** Cross-session experiment comparison results.

Dataset	Method	Year	Average Acc (%)	Kappa	Std
BCIC IV 2a	DeepConvNet	2017	66.87	0.59	15.03
	EEGNet	2018	68.18	0.57	14.25
	Sinc-ShallowNet	2020	73.34	0.65	12.80
	G-CRAM	2020	72.53	0.64	12.35
	BiLSTM-GCN	2022	73.65	0.67	12.06
	EEG-Conformer	2023	75.37	0.69	12.74
	MGCANet	—	**78.26**	**0.70**	**10.50**
OpenBMI	DeepConvNet	2017	60.08	0.31	14.95
	EEGNet	2018	68.17	0.39	13.06
	Sinc-ShallowNet	2020	68.64	0.36	13.90
	G-CRAM	2020	68.05	0.36	14.21
	BiLSTM-GCN	2022	67.92	0.39	15.14
	EEG-Conformer	2023	66.45	0.35	14.32
	MGCANet	—	**73.68**	**0.45**	**12.82**

**Table 2:** The result of the ablation study.

Dataset	w/o P-View (%)	w/o F-View (%)	w/o Res (%)	w/o Awf (%)	w/o MHA (%)	MGCANet
BCIC IV 2a	74.83	74.67	75.21	77.39	75.60	**78.26**
OpenBMI	69.95	70.39	69.72	72.65	71.14	**73.68**

**Table 3:** The results of parameter selection.

Parameter	Value	BCIC IV 2a (%)	OpenBMI (%)
K	1	74.34	71.17
	2	76.58	71.45
	3	**78.26**	**73.68**
	4	74.03	70.92
Number of layers	1	75.18	70.60
	2	76.72	71.61
	3	**78.26**	**73.68**
	4	72.51	69.24
Numbers of Heads	1	77.65	72.75
	4	77.73	73.16
	6	78.04	73.51
	8	**78.26**	**73.68**
	10	77.48	72.24
Max norm	0.1	73.63	71.90
	0.2	75.84	72.44
	0.5	**78.26**	**73.68**
	1.0	73.19	71.07

**Table 4:** The results of the ablation study of the adaptive-weighted fusion.

Method	Fusion	BCIC IV 2a (%)	OpenBMI (%)
MGCANet	add	76.48	72.24
	concat	77.25	72.85
	proposed	**78.26**	**73.68**

**Table 5:** The results of the different attention methods.

Dataset	Method	Average Acc (%)	Std (%)
BCIC IV 2a	SE	73.95	15.76
	ECA	76.67	11.08
	SA	77.20	12.28
	MHA	**78.26**	**10.50**
OpenBMI	SE	70.24	16.03
	ECA	72.52	13.24
	SA	71.43	12.95
	MHA	**73.68**	**12.82**

## Data Availability

The data presented in this study are openly available at the following URL/DOI: https://bbci.de/competition/iv/ accessed on 10 May 2023.
